# Supplementary material for: Cognitive–Behavioral Profile in Pediatric Patients with Syndrome 5p-; Genotype–Phenotype Correlationships
Source: Genes (Basel). 2023 Aug 15;14(8):1628. doi: 10.3390/genes14081628 (PMC10454038; doi:10.3390/genes14081628)

Table S5. ***Supplemental data.*** *Coordinates of genetic alterations (CNVs).*

	Deletion				Duplication			
Individual	Chromosome	Initial coordinate	Final coordinate	Size (Mb)	Chromosome	Initial coordinate	Final coordinate	Size (Mb)
1	5p15.33-p15.1	25328	17981563	17.98	Xp22.33	169805	2123990	.212
2	5p15.33-p14.3	25328	22446214	22.44	10q25.3-q26.3	115402474	135434319	20.08
3	5p15.33-p15.2	25328	12995938	12.99				
4	5p15.33-p14.1	25328	28779357	28.77				
5	5p15.33-p14.3	25328	22956970	22.95				
6	5p15.33-p15.31	25328	9438756	9.43	Xq28	154933691	155236712	3.72
7	5p15.33-p13.2<br>5p13.2	25328<br>34602654	34602269<br>36816661	34.6<br>2.21*				
8	5p15.33-p13.3	25328	31853346	31.85				
9	5p15.33-p13.2	25328	35015297	35.02				
10	5p15.33-p14.1	25328	25290077	25.3				
11	5p15.32-p15.1	4928318	15418957	10.49				
12	5p15.33-p14.2	25328	24430251	24.43				
13	5p15.33-p14.1	25328	28783716	28.78				
14	5p15.33-p15.32	25328	4938756	4.94				
15	5p15.33-p13.3	25328	34986724	34.98				

Individual	Deletion				Duplication			
	Chromosome	Initial coordinate	Final coordinate	Size (Mb)	Chromosome	Initial coordinate	Final coordinate	Size (Mb)
16	5p15.33-p15.1	25328	17665529	17.6	12p11.21	32875287	33056330	0.18
17	5p15.33-p14.1	25328	25027051	25.02				
18	5p15.33-p15.1	25328	15913112	15.91	8p23.3p-23.1	176617	11860710	11.86
19	5p15.33-p14.1	25328	25396006	25.40	5p14.1	25409917	28435493	3.02
20	5p15.33-p15.1	25328	15808138	15.81				
21	5p15.33-p15.2	25328	12978580	12.08	10q-26.11-q26.3	121556072	135425341	13.87
22	5p15.33-p15.2	25328	11037420	11.037				
23	5p15.33-p15-32	25328	4356789	4.36	5p15.33-p15.32<br>5p15.31	4355708<br>6325532	4969019<br>6642356	0.60<br>0.30
24	5p15.33-p14.1	25328	27108052	27.10				
25	5p15.33-p14.3	25328	21872896	21.88	9p24.3-p22.1	46587	19713500	19.7
26	5p15.33-p14.3	25328	22658970	22.65	8p23.2-p11.23	2061877	34908297	34.94
27	5p15.33-p15.2	25328	14360436	14.36				
28	5p15.33-p13.3	25328	29485091	29.48				
29	5p15.33-p13.3	25328	29292854	29.29	1p13.1-p12	117594464	117989275	0.38
30	5p15.33-p13.3	25328	32130401	32.13				
31	5p15.33-p14.1	25328	27708038	27.71	18p11.32	13034	2656248	2.65
32	5p15.33-p14.1	25328	28147535	28.15				

	Deletion				Duplication			
Individual	Chromosome	Initial coordinate	Final coordinate	Size (Mb)	Chromosome	Initial coordinate	Final coordinate	Size (Mb)
33	5p15.33-p14.1	25328	26622073	26.62	5p13.3-p13.2	26695268	34019038	7.67
34	5p15.33-p14.1	25328	28796749	28.79				
35	5p15.33-p15.1	560000	17509888	16.95				
36	5p15.33-p14.1	25328	25821865	25.82				
37	5p15.33-p14.3	25328	21504581	21.50	8p23.3-p23.2<br>22q11.21	164984<br>25661725	752709<br>25914593	0.75<br>0.25
38	5p15.33-p15.32	2656248	4610206	4.61	5q35.1<br>5q35.1-q35.3	169708691<br>171656863	169893751<br>180693344	0.18<br>9.03
39	5p15.33-p15.1	25328	15922302	15.92				
40	5p15.33-p14.2	25328	24438467	24.43				
41	5p15.33-p14.1	25328	25135494	25.13	11q22.1	100578089	100870339	0.29
42	5p15.33-p15.1	25328	15022112	15.02	9p24.3-p21.3	162931	23232287	23.24
43	5p15.33-p14.1	25328	28464893	28.46	18p11.32-p11.31	141896	6785383	6.84
44	5p15.33-p14.2	25328	24247673	24.24				
45	5p15.33-p15.1	25328	17704161	17.70	5p14.3	19970119	20370847	0.40

* Mosaicism

Figure 1 ***Supplemental data.*** *Graphic illustration of the 5p deletions found in the cohort*

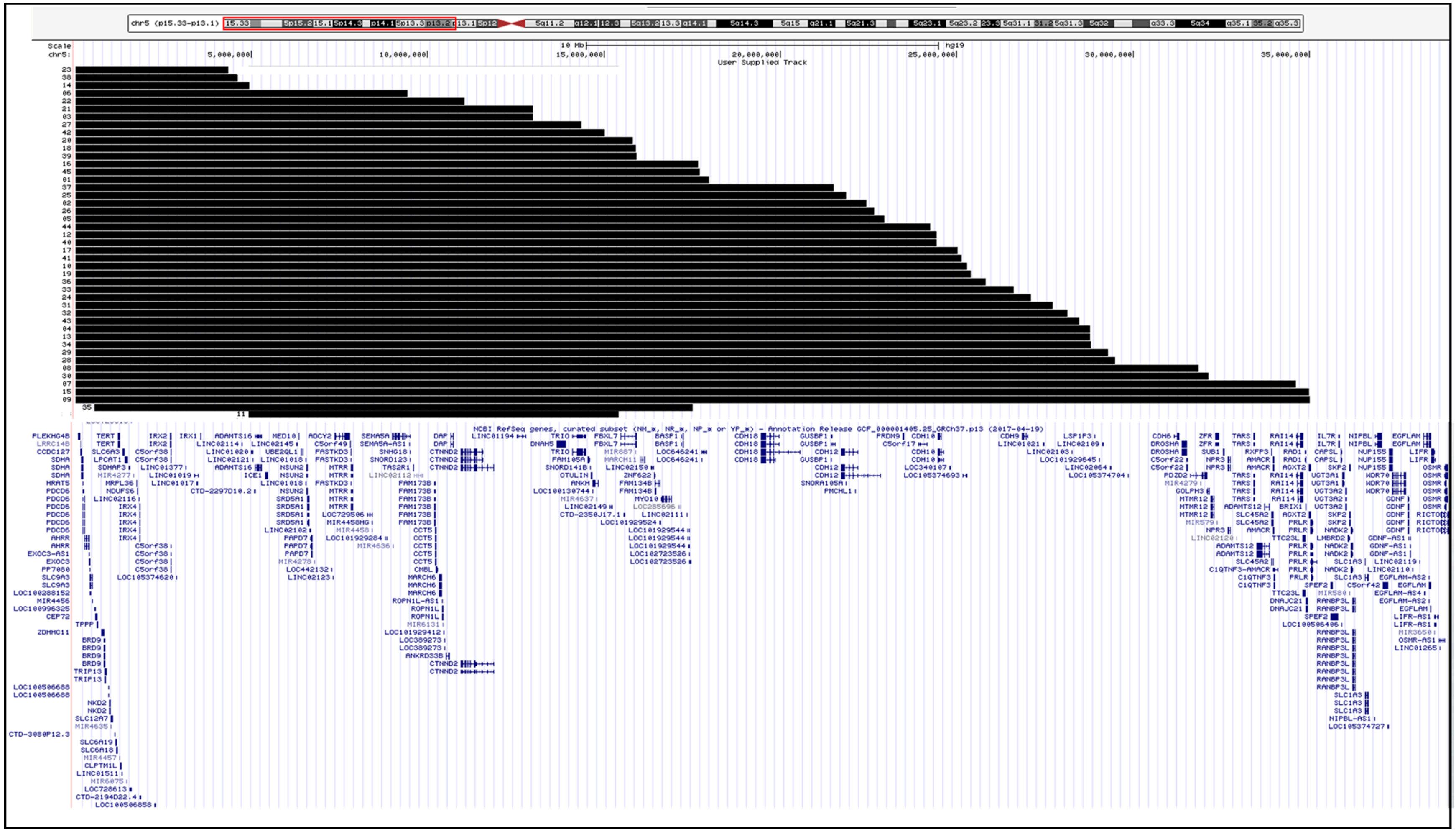

Supplement: Supplementary file 1 [file genes-14-01628-s001.zip › Table S5 y Figure 1 suplem.pdf]
