# Supplementary material for: Cognitive–Behavioral Profile in Pediatric Patients with Syndrome 5p-; Genotype–Phenotype Correlationships
Source: Genes (Basel). 2023 Aug 15;14(8):1628. doi: 10.3390/genes14081628 (PMC10454038; doi:10.3390/genes14081628)
Supplement: Supplementary file 1 [file genes-14-01628-s001.zip › Table S7-S9 suplemental data.pdf]

Tabla S7. Supplemental data. *Pearson correlations between cognitive variables and BPI-01 results*

	PERS	ADAP	MTGR	MTF	MOT	RECP	EXP	LENG	COG	TOT	AUTFR	AUTGR	ESTFR	ESTGR	AGRFR	AGRGR	TOTFR	TOTGR
PERS	-	,960**	,918**	,859**	,928**	,811**	,858**	,866**	,858**	,971**	-,128	-,125	**-,369***	**-,337***	-,235	-,267	-,286	-,271
ADAP		-	,944**	,882**	,948**	,835**	,848**	,869**	,883**	,976**	-,201	-,179	**-,396****	**-,350***	-,280	**-,307***	**-,344***	**-,315***
MTGR			-	,904**	,987**	,842**	,833**	,862**	,881**	,961**	-,085	-,090	**-,340***	-,278	-,162	-,165	-,233	-,202
MTF				-	,943**	,841**	,836**	,864**	,849**	,916**	-,074	-,080	**-,307***	-,259	-,171	-,209	-,218	-,203
MOT					-	,872**	,889**	,910**	,913**	,979**	-,122	-,122	**-,354***	**-,294***	-,202	-,204	-,268	-,235
RECP						-	,877**	,953**	,856**	,887**	-,269	-,262	**-,382****	**-,319***	**-,305***	**-,311***	**-,377***	**-,344***
EXP							-	,981**	,907**	,920**	-,279	-,270	**-,391****	**-,337***	**-,325***	**-,313***	**-,391****	**-,356***
LENG								-	,914**	,935**	-,283	-,275	**-,399****	**-,340***	**-,327***	**-,322***	**-,397****	**-,362***
COG									-	,938**	-,256	-,230	**-,397****	**-,349***	**-,358***	**-,338***	**-,391****	**-,348***
TOT										-	-,187	-,177	**-,393****	**-,344***	-,276	-,288	**-,336***	**-,305***
AUTFR											-	,915**	,547**	,571**	,689**	,593**	,897**	,847**
AUTGR												-	,569**	,599**	,576**	,558**	,836**	,891**
ESTFR													-	,955**	,469**	,534**	,825**	,812**
ESTGR														-	,491**	,558**	,822**	,852**
AGRFR															-	,873**	,799**	,727**
AGRGR																-	,748**	,781**
TOTFR																	-	,952**
TOTGR																		-

BPI-01: COGNITIVE: PERS = personal area; ADAP = adaptive area; MTGR = gross motor area; MTF = fine motor area; MOT = total motor; RECP = receptive language area; EXP = expressive language area; COG = cognitive area; TOT = total. ESTFR: stereotyped behavior frequency; ESTGR: stereotyped behavior severity; AGRFR aggressive behavior frequency; AGRGR aggressive behavior severity; TOTFR = total frequency; TOTGR: total severity. *The correlation is significant at the level 0.05 (bilateral). ** The correlation is significant at the level 0.01 (bilateral).

Tabla 8. Supplemental data. *Pearson correlations between cognitive variables and RBQ results*

	PERS	ADAP	MTGR	MTF	MOT	RECP	EXP	LENG	COG	TOT	CEST	CCOM	PLIM	DREP	IMON	RBQ
PERS	-	,960**	,918**	,859**	,928**	,811**	,858**	,866**	,858**	,971**	**-,378***	**,347***	,264	-	,255	,055
ADAP		-	,944**	,882**	,948**	,835**	,848**	,869**	,883**	,976**	**-,384****	**,354***	,204	-	,234	,023
MTGR			-	,904**	,987**	,842**	,833**	,862**	,881**	,961**	**-,344***	**,344***	**,309***	-	,293	,104
MTF				-	,943**	,841**	,836**	,864**	,849**	,916**	**-,309***	**,396****	,279	-	**,304***	,134
MOT					-	,872**	,889**	,910**	,913**	,979**	**-,368***	**,359***	**,298***	-	**,313***	,096
RECP						-	,877**	,953**	,856**	,887**	**-,445****	**,346***	,174	-	,288	-,009
EXP							-	,981**	,907**	,920**	**-,446****	,288	,195	-	,280	-,019
LENG								-	,914**	,935**	**-,459****	**,320***	,192	-	,291	-,016
COG									-	,938**	**-,461****	**,406****	,265	-	**,298***	,037
TOT										-	**-,415****	**,370***	,260	-	,286	,048
CEST											-	-,082	-,255	-	-,002	,663**
CCOM												-	,372*	-	,396**	,489**
PLIM													-	-	,389**	,763**
DREP														-	-	-
IMON															1	,577**
RBQ																-

RBQ: COGNITIVE: PERS = personal area; ADAP = adaptive area; MTGR = gross motor area; MTF = fine motor area; MOT = total motor; RECP = receptive language area; EXP = expressive language area; COG = cognitive area; TOT = Total. CEST: stereotyped behavior; CCOM: compulsive behavior; PLIM: Limited Preferences; DREP = repetitive speech; MON = Insistency on monotony; *The correlation is significant at the level 0.05 (bilateral). ** The correlation is significant at the level 0.01 (bilateral).

Tabla 9. Supplemental data. *Pearson correlations between cognitive variables and DASH-II results*

	PERS	ADAP	MTGR	MTF	MOT	RECP	EXP	LENG	COG	TOT	IMP FR	IMP GR	ORG FR	ORG GR	HUM FR	HUM GR	MAN FR	MAN GR	AUT FR	AUT GR	EST FR	ESTGR	AGR FR	AGR GR	PSUFR	PSU GR	TOT FR	TOT GR
PERS	-	,960**	,918**	,859**	,928**	,811**	,858**	,866**	,858**	,971**	-,2	-,161	-,085	-,059	-,155	-,211	-,165	-,2	-,139	-,084	**-,460****	**-,386****	-,007	-,082	**-,400****	**-,424****	**-,362***	**-,296***
ADAP		-	,944**	,882**	,948**	,835**	,848**	,869**	,883**	,976**	-,211	-,153	-,117	-,097	-,2	-,26	-,259	**-296***	-,178	-,135	**-,519****	**-,446****	-,043	-,124	**-,461****	**-,491****	**-,429****	**-,372***
MTGR			-	,904**	,987**	,842**	,833**	,862**	,881**	,961**	-,165	-,117	-,053	-,051	-,152	-,226	-,232	**-,325***	-,114	-,089	**-,467****	**-,417****	,03	-,049	**-,455****	**-,498****	**-,348***	**-,320***
MTF				-	,943**	,841**	,836**	,864**	,849**	,916**	-,186	-,155	-,154	-,055	-,174	-,259	-,262	-,247	-,082	-,056	**-,447****	**-,380***	,04	-,032	**-,365***	**-,432****	**-,344***	-,277
MOT					-	,872**	,889**	,910**	,913**	,979**	-,189	-,145	-,084	-,061	-,153	-,231	-,251	**-,311***	-,087	-,057	**-,475****	**-,417****	,018	-,063	**-,444****	**-,491****	**-,350***	**-,314***
RECP						-	,877**	,953**	,856**	,887**	-,241	-,201	-,112	-,081	-,169	-,229	**,314***	**-,316***	-,156	-,125	**-,482****	**-,433****	-,193	-,251	**-,422****	**-,444****	**-,426****	**-,404****
EXP							-	,981**	,907**	,920**	-,178	-,155	-,138	-,062	-,202	-,242	**-,311***	**-,297***	-,046	-,011	**-,475****	**-,417****	-,099	-,181	**-,412****	**-,405****	**-,381****	**-,324***
LENG								-	,914**	,935**	-,209	-,178	-,132	-,071	-,195	-,244	**-,322***	**-,314***	-,092	-,057	**-,492****	**-,436****	-,14	-,215	**-,429****	**-,434****	**-,411****	**-,366***
COG									-	,938**	-,141	-,099	-,064	-,068	-,154	-,216	-,274	**-,368***	,029	,026	**-,508****	**-,488****	,006	-,132	**-,411****	**-,461****	**-,339***	**-,321***
TOT										-	-,199	-,154	-,098	-,072	-,175	-,239	-,25	**-,296***	-,107	-,07	**-,505****	**-,442****	-,025	-,115	**-,444****	**-,477****	**-,389****	**-,341***
IMPFR											-	,895**	,209	,012	,386**	,500**	,225	-,042	,373*	,130	,403**	,107	,221	,203	,267	,214	,493**	,369*
IMPGR												-	,303*	,039	,225	,324*	,157	-,133	,325*	,130	,272	,070	,243	,220	,101	,076	,379*	,324*
ORGFR													-	,309*	,374*	,349*	,602**	-,133	-,049	-,183	,108	,004	,134	,046	,198	,114	,345*	,052
ORGGR														-	,166	,117	,252	,078	-,083	-,045	-,008	,046	,256	,166	,068	,004	,108	,106
HUMFR															-	,950**	,577**	,129	,226	,018	,319*	,112	,189	,137	,686**	,437**	,679**	,327*
HUMGR																-	,589**	,185	,281	,065	,406**	,183	,124	,077	,673**	,528**	,698**	,386**
MANFR																	-	,570**	,268	,150	,467**	,371*	,428**	,399**	,606**	,537**	,716**	,544**
MANGR																		-	,323*	,410**	,472**	,547**	,333*	,425**	,516**	,596**	,451**	,655**
AUTFR																			-	,870**	,646**	,539**	,487**	,454**	,233	,244	,695**	,746**
AUTGR																				-	,491**	,639**	,470**	,496**	,168	,242	,495**	,787**
ESTFR																					-	,851**	,316*	,415**	,403**	,402**	,805**	,736**
ESTGR																						-	,338*	,485**	,315*	,384**	,624**	,798**
AGRFR																							-	,940**	,263	,170	,502**	,677**
AGRGR																								-	,305*	,247	,504**	,755**
PSUFR																									-	,881**	,629**	,565**
PSUGR																										-	,496**	,598**
TOTFR																											-	,779**
TOTGR																												-

DASH-II: IMPFR = impulses, frequency; IMPGR = impulses, severity; ORGFR = organic, frequency; ORGGR = organic, severity; ANSFR = anxiety, frequency; ANSGR= anxiety, severity; HUMFR = mood, frequency; HUMGR = mood, severity; MANFR = mania, frequency; MANGR = mania, severity; AUTFR = autism, frequency; AUTGR = autism, severity; ESQFR = schizophrenia, frequency; ESQGR = schizophrenia, severity; ESTFR = stereotypes, frequency; ESTGR = stereotypes, severity; AUTFR = self-harm, frequency; AUTGR = self-harm, severity; PSUFR = sleep problems; frequency; PSUGR = sleep problems, severity; TOTFR = total frequency; TOTGR = total severity. COGNITIVE: PERS = personal area; ADAP = adaptive area; MTGR = gross motor area; MTF = fine motor area; MOT = Total motor; RECP = receptive language area; EXP = expressive language area; COG = cognitive area; TOT = Total. *The correlation is significant at the level 0.05 (bilateral). ** The correlation is significant at the level 0.01 (bilateral).
