# Supplementary material for: Cognitive–Behavioral Profile in Pediatric Patients with Syndrome 5p-; Genotype–Phenotype Correlationships
Source: Genes (Basel). 2023 Aug 15;14(8):1628. doi: 10.3390/genes14081628 (PMC10454038; doi:10.3390/genes14081628)
Supplement: Supplementary file 1 [file genes-14-01628-s001.zip › Tables S1 S2 S3 S4 S6 Supplemental data.pdf]

Table S1 Supplemental data. *Distribution of the total sample by age and sex*

Age (years)	Men	Women	%
0	1	0	2,2
1	1	2	6,7
2	1	3	8,9
3	2	5	15,5
4	1	5	13,3
5	1	0	2,2
6	0	2	4,4
7	0	1	2,2
8	3	5	17,8
9	0	3	6,7
10	3	1	8,9
11	0	1	2,2
12	0	2	4,4
13	0	2	4,4
TOTAL	13 (29%)	32 (71%)	

Table S2 Supplemental data. Neonatal data of this cohort

	Men			Women			Total		
	Mean	Range	*SD*	Mean	Range	*SD*	Mean	Range	*SD*
Gestational Weeks	37.82	32-41.6	2.46	38.37	31-42	2.74	38.2	31-42	2.64
Weight (g)	2,757.7	1,170-4,500	784.55	2,431.97	1,200-3,800	600.95	2,526.07	1,170-4,500	666.78
Height (cm)	46.96	39-52	3.84	45.68	39-51	3.04	46.05	39-52	3.30
OFC (cm)	32.23	29-37	2.63	31.75	27-37	2.12	31.89	27-37	2.21

SD*,* standard deviation.

Table S3 Supplemental data. *Dysmorphic features in the cohort*

	Men		Women		Total	
	N	%	N	%	N	%
Rounded face	4	30.8	18	56.2	22	48.9
Large face	5	38.5	5	15.6	10	22.2

	Men		Women		Total	
	N	%	N	%	N	%
Well-pronounced supercilliary arches	1	7.7	3	9.4	4	8.88
Alteración en los pabellones auriculares	8	61.5	18	56.2	26	57.8
Epicanthus	9	69.2	17	53.1	26	57.8
Hypertelorism	9	69.2	21	65.6	30	66.7
Downward slanting palpebral fissures	3	23.1	8	25	11	24.4
Wide nasal bridge	9	69.2	16	50	25	55.6
Short philtrum	1	7.7	3	9.4	4	8.88
Cleft palate	0	-	1	3.1	1	2.22
Ogival palate	2	15.4	2	6.2	4	8.88
Big mouth	6	46.1	3	9.4	9	20
Dental anomalies	6	46.1	21	65.5	27	60
Micrognathia	7	53.8	16	50	23	51.1
Short neck	0	-	3	9.4	3	20

Table S4 Supplemental data*. Co-morbidity features in the cohort*

	Frequency	%	Type of alterations
NEUROLOGICALS			
- Microcephaly	41	91.1	
- other anomalies	13	28.9	Facial paralysis, corpus callosum disorders, frontal horn ectasia, cerebellar tonsil herniation, brainstem hypoplasia, dilated ventricular system, cysts, hydrocephalus
- Hypotonía	38	84.4	
- Hypertonía	4	8.9	
- Epilepsy	3	6.7	
HEART DISEASE	19	42,2	Congenital heart disease, patent ductus, patent foramen ovale (POF), atrial septal defect, ventricular septal

	Frequency	%	Type of alterations
			defect, ventricular hypertrophy, murmur
DIGESTIVE DISORDERS			
- malformations	20	44.4	Anal fissure, rectal muscle defect, inverted duodenal atresia, inguinal hernia, umbilical hernia, congenital hydronephrosis hemorrhoids
- Reflux	20	44.4	
- Other	18	40	Vomiting, digestive spasms, esophagitis, severe chronic constipation
RESPIRATORY ATERATIONS			
-Alterations in the larynx	12	26.7	
- Recurrent respiratory infections	9	20	Bronchiolitis, Asthma
- Respiratory problems	23	51,1	Vegetations, tonsils, sleep apnea
MUSCULOSKELETAL ALTERATIONS			
- Dental	27	60	Malformed teeth, misalignment, poor occlusion, caries
- hands malformations	20	44.4	Growth anomalies, syndactyly
- Foot malformations	10	22.2	Flatfoot, valgus foot, clubfoot, alt. Growth, syndactyly
- Scoliosis	17	37.8	
- Articular Hyperlaxitud	21	46.7	
RENAL	7	15.5	Renal malformation, cysts, nephrocalciosis, renal reflux, renal dilation, hemangiomas, hydronephrosis, nonspecific incontinence
GENITALS	9	21.4	Microrchidism, cyptorchidism, synechia of labia minora.
SENSORIALs			
- Opthalmological	25	55,5	Strabismus, myopia, farsightedness, astigmatism, nystagmus, convergence insufficiency, pigment epithelial atrophy, retinal hemorrhage
- Hearing	24	53,3	Hypersensitivity to noise, hearing loss, auditory polyneuropathy,
- Others	7	15,5	Dermatological: dermatitis, atopic skin, rosacea; metabolic alterations

Tabla S6 of Supplemental data. *Sample distribution according to motor milestones*

Motor milestones	0-2 yrs.	3-4 yrs.	4-6 yrs.	6-10 yrs.	10-11 yrs.	11-14 yrs.
N	8	7	7	14	4	5
Keeps sitting without support	1	5	6	14	4	5
Sits alone	0	5	6	14	4	5
Stands with help	0	5	6	14	4	5
Walks with help	0	5	6	12	3	4
Walks unaided	0	5	6	12	3	4
Runs 3 meters	0	2	2	6	3	3
Jump with feet together	0	2	1	4	1	3
Alternating up and down stairs	0	1	0	4	1	3
Ulnar palmar pressure	6	7	7	14	4	5
Digital partial pressure	0	4	6	14	4	5
Top clamp	0	1	2	7	2	3
Build a 2-block tower	0	1	5	7	3	4
String 4 large beads	0	1	1	4	2	2
Copying simple strokes	0	1	2	7	2	3
